# Don’t neglect the non-thrombotic manifestations of antiphospholipid syndrome in children – autoimmune hemolytic anemia and myocarditis: a case report and literature review

**DOI:** 10.3389/fimmu.2026.1724748

**Published:** 2026-01-30

**Authors:** Wanlin Cui, Yuming Sun, Danyang Zhao, Masumeh Nozzari Varkani, Guan Wang, Xiaoqing Wen, Mingyue Shi, Xiaohan Wang, Xinyi Zhang, Hongkun Jiang

**Affiliations:** 1 Department of Pediatrics, The First Hospital of China Medical University, Shenyang, Liaoning, China; 2 Department of Radiology, The First Hospital of China Medical University, Shenyang, Liaoning, China

**Keywords:** antiphospholipid syndrome, case report, hemolytic anemia, myocarditis, pediatric immunology

## Abstract

Antiphospholipid Syndrome (APS) is a systemic autoimmune disorder characterized by persistent antiphospholipid antibodies, associated with thrombosis or adverse pregnancy outcomes. Although non-thrombotic manifestations are less common than thrombotic events, they play an increasingly important role in diagnosis and disease progression, particularly in pediatric APS, and may interact with each other. Early recognition and management of these symptoms are crucial for patient prognosis. We present a case of 13-year-old male child presenting as autoimmune hemolytic anemia (AIHA) and myocarditis, with positive antiphospholipid antibodies (aPL). The patient showed significant improvement after combination therapy with corticosteroids, prophylactic low molecular weight heparin and aspirin. In addition, we conducted a comprehensive literature review on APS in conjunction with hemolytic anemia and cardiac complications, and found that in 171 APS cases with hemolytic anemia, 35 of them had cardiac complications. There were 8 cases of myocardial infarction (8/171,4.7%) and 3 cases of myocarditis (3/171, 1.8%). Compared with AIHA, the incidence of cardiopathy was significantly higher in microvascular hemolytic anemia (MAHA) (p = 0.020). Among 13 cases that recorded the time window from hemolytic anemia to cardiac symptoms, 92.3% of them developed cardiac complications within one year after the onset of hemolytic anemia, typically within a median interval of 3 months (ranging from 5 days to 2 years). Notably, 28.6% of the 35 cases reviewed involved children under 18, with nearly half presenting hemolytic anemia as the initial symptom. This case underscores APS patients with hemolytic anemia, particularly MAHA, require early intervention and timely cardiac follow-up. Given the current lack of definitive classification and treatment criteria for pediatric APS, future guidelines should incorporate the significance of non-thrombotic manifestations and emphasize early management strategies.

## 1 Introduction

Antiphospholipid syndrome (APS) is a systemic autoimmune disease characterized by recurrent vascular thrombosis or pregnancy complications, with persistent positivity for antiphospholipid antibodies (aPL) (1). It can present as a primary condition, called primary APS or can be secondary to other autoimmune diseases, commonly seen in systemic lupus erythematosus (SLE) (2, 3). Pediatric antiphospholipid syndrome (Ped-APS) is relatively rare, and the prevalence of secondary APS is slightly higher than that of primary APS (4, 5).

In addition to the common arterial and venous thrombosis, APS can present as microthrombosis or a variety of non-thrombotic manifestations (6), such as thrombocytopenia, autoimmune hemolytic anemia (AIHA), cardiac valve disease, livedo reticularis, kidney disease, chorea, migraine, and cognitive dysfunction (7, 8), and these manifestations may interact with each other (9). AIHA is typically caused by immune-mediated destruction of red blood cells (10). Notably, patients who initially present with hemolytic anemia have a significantly increased incidence of subsequent serious events (11). Myocarditis is less common in APS but may cause severe consequences, potentially resulting from microvascular thrombosis or direct immune-mediated myocardial injury (12, 13). It is particularly important to note that Ped-APS is not common, there is no pediatric-specific classification criteria, and diagnosis often relies on “adult criteria and the SHARE Initiative” (14, 15). However, compared to adults, Ped-APS is more likely to present with non-thrombotic symptoms, sometimes even as the initial symptom, which can be easily overlooked and lead to delayed diagnosis (4, 16). Therefore, early recognition and appropriate anticoagulation therapy are crucial for preventing the morbidity and mortality associated with APS.

Herein, we present a case of a 13-year-old male who was diagnosed with primary APS associated with AIHA and myocarditis. Additionally, we conducted a comprehensive literature review on APS in conjunction with hemolytic anemia and cardiac complications, aiming to explore their clinical features and to investigate any potential associations between hemolytic anemia and cardiac symptoms in the context of APS. This review seeks to enhance the understanding of the diagnosis and management of these associated complications.

## 2 Case presentation

A 13-year-old male patient presented to our department with symptoms of fever and fatigue for 5 days. Blood tests at a local hospital indicated a hemoglobin (Hb) level of 43 g/L (normal 120-165g/L). The child had no respiratory or gastrointestinal symptoms, no epistaxis, no gross hematuria, and no melena. His Hb level was normal one month prior to admission. He had no previous medical problems with no family history of genetic diseases.

Laboratory tests showed a significant decrease in Hb to 37 g/L and an elevated reticulocyte count of 93.5 × 10^9^/L (normal 24-84 × 10^9^/L). Direct Coombs test revealed positivity for C3 and polyspecific antibodies. Activated partial thromboplastin time (APTT) was significantly prolonged at 58.6 seconds (normal 25–35 seconds) and could not be corrected. The platelet count dropped to 80 × 10^9^/L. The lupus anticoagulant (LAC) assay detected a positive result. Additionally, standardized enzyme-linked immunosorbent assay (ELISA) testing revealed moderate or high positivity for both anticardiolipin (aCL) and anti-β_2_-glycoprotein I (anti-β_2_GPI) antibodies (IgG and IgM). Rheumatological and immunological tests, including antinuclear antibody, anti-double-stranded DNA and anti-Smith antibodies, were both negative, with normal C3 and C4 levels. No evidence of thrombosis.

**Figure 1:**
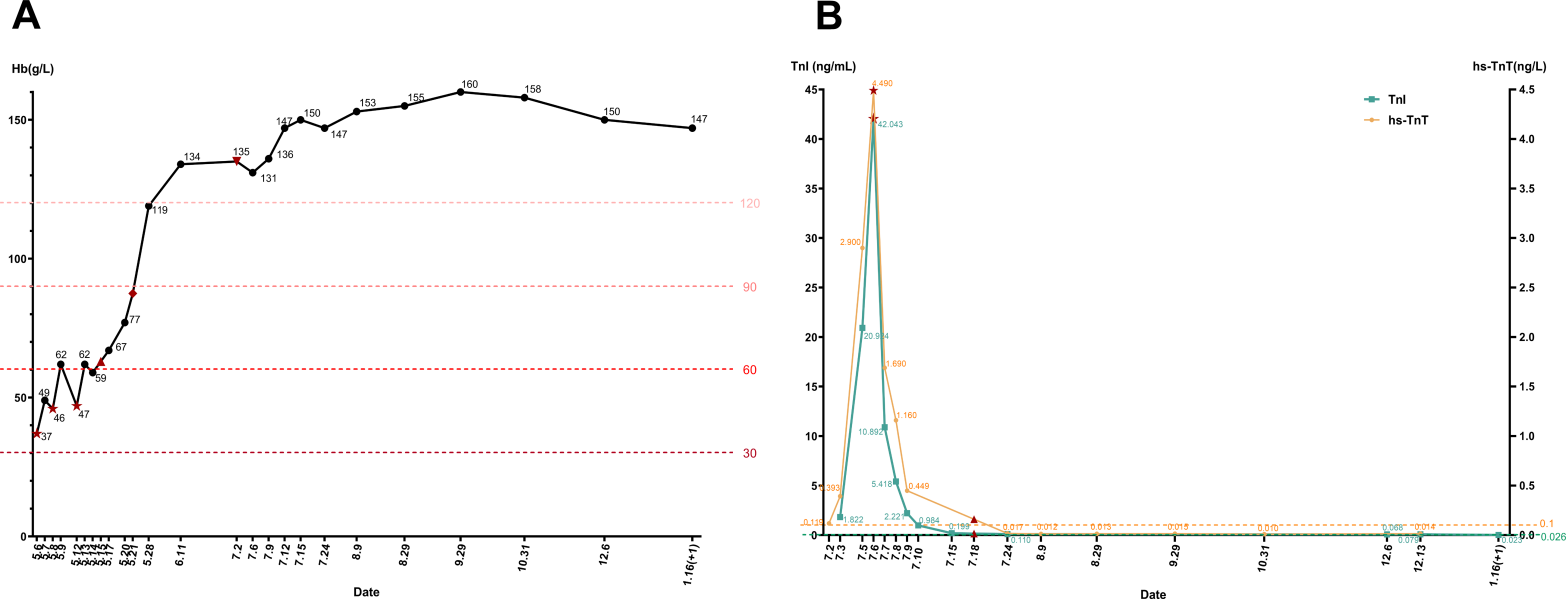
**(A)** The line chart shows the hemoglobin (Hb) changes of the patient during the whole course of treatment. The patient presented with severe anemia upon initial admission, and corticosteroid treatment quickly corrected the anemia. ★ The patient was transfused with Leukocyte-reduced Red Blood Cells suspension on May 6, May 8, and May 12, and Hb briefly increased after infusion. ▴ On May 15, the patient was prescribed prednisone and prophylactic low molecular weight heparin therapy. ♦ On May 21, the patient was discharged and prescribed oral prednisone following discharge. ▾ On July 2, the patient discontinued corticosteroids. **(B)** The line chart shows changes in troponin I (TnI) and high-sensitivity troponin T (hs-TnT) in patients throughout the course of treatment. When the patient was admitted to hospital for the second time on July 2, the laboratory examination showed that TnI and hs-TnT were particular higher than normal. With the onset of treatment, the patient’s TnI and hs-TnT decreased to normal. ★ On July 6, the patient was prescribed corticosteroid, aspirin, prophylactic low molecular weight heparin therapy, TnI and hs-TnT decreased significantly after drug administration. ▴ On July 18, the patient was discharged, and continued with oral prednisone and aspirin.

Based on these findings, despite the presence of three positive aPL, the absence of clinical symptoms meeting APS classification criteria led us to provide personalized treatment for the patient. The patient was initiated on a combination therapy including prednisone (20 mg per dose, three times daily) and low-molecular weight heparin (4100 IU nadroparin calcium, once daily). Two weeks later, Hb levels rapidly achieved to normal (**Figure 1A**). One month later, prophylactic low molecular weight heparin was discontinued, and the dose of corticosteroid was gradually tapered and discontinued after two months.

**Figure 2:**
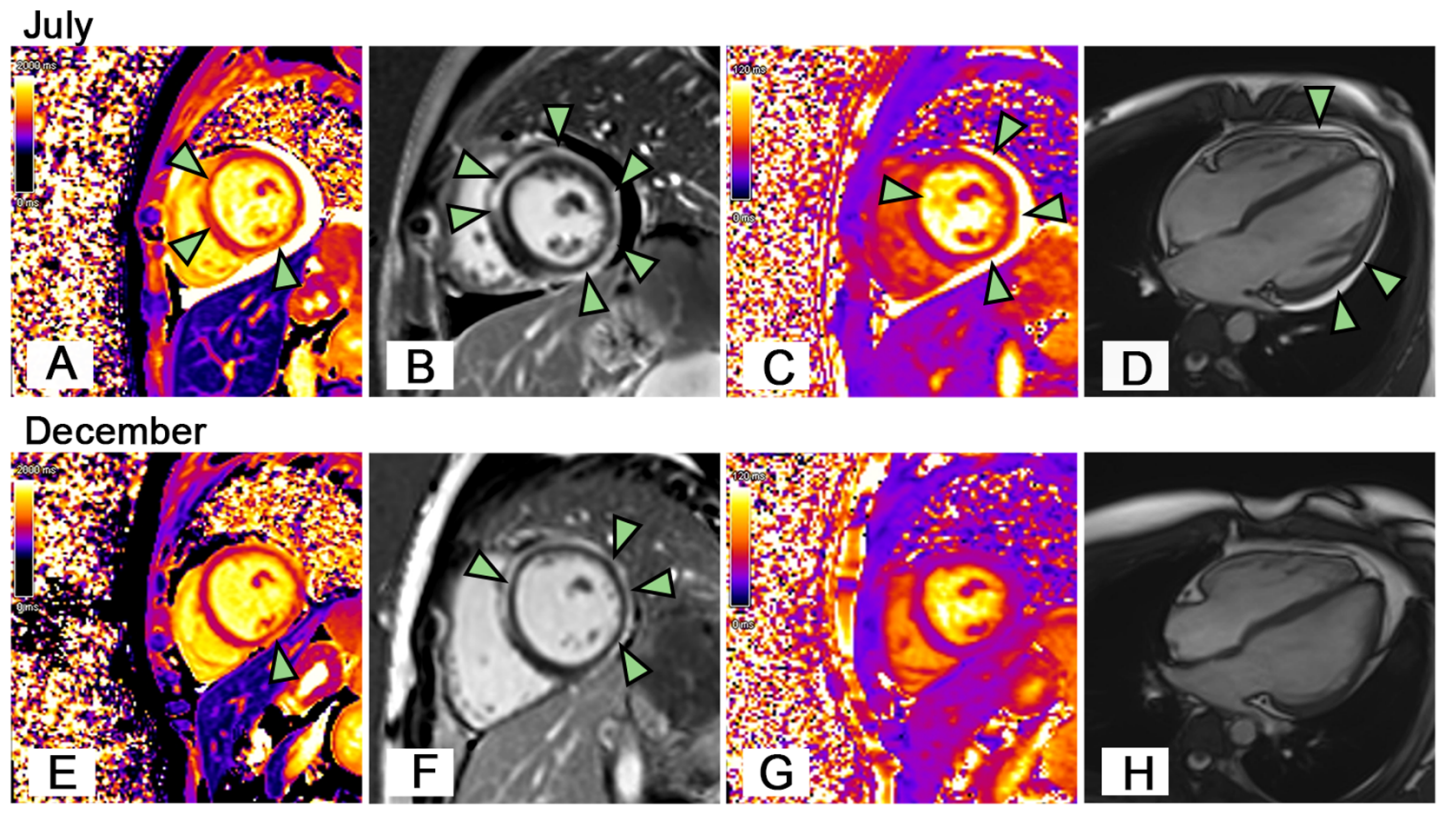
Cardiac Magnetic Resonance Imaging. On July **(A)** The patient’s short-axis view showed local increases in T1 values in the anterior septum and inferior wall of the left ventricle (arrowheads). **(B)** The patient’s LGE image showed delayed subepicardial enhancement of the left ventricle, including the anterior septum, anterior wall, lateral wall, posterior wall, and inferior wall (arrowheads). **(C)** The patient’s short-axis view showed increased T2 values in the left ventricular myocardium (edema), especially in the anterior septum, inferior, posterior, and lateral walls (arrowheads). **(D)** The patient had pericardial effusion (arrowheads). On December **(E)** The T1 value of the anterior septum of the left ventricle decreased to normal, while the local T1 value of the inferior wall was still increased (arrowhead). **(F)** LGE image showed the delayed enhancement of the left ventricular subepicardium persisted (arrowheads). **(G)** The overall left ventricular T2 value decreased to normal. **(H)** The pericardial effusion disappeared.

However, during the follow-up two weeks later, it was found that the troponin I (TnI) level increased to 1.822 ng/mL (normal <0.026 ng/mL), and the high-sensitivity troponin T (hs-TnT) level increased to 0.119 ng/L (normal <0.1 ng/L) (**Figure 1B**). Electrocardiogram (ECG) indicated sinus arrhythmia. There were no symptoms of chest discomfort, pain, palpitations, or fatigue. Pathogen detection was negative, ruling out bacterial or viral infections as causes of myocarditis. Subsequent laboratory evaluations showed another significant prolongation of APTT, with LAC was still positive, and according to ELISA, aCL and anti-β2GPl (lgG and lgM) still showing moderate to high positivity. Imaging studies, including echocardiography and multisite CTA, showed no thrombotic lesions. Cardiovascular magnetic resonance (CMR) with contrast suggested myocarditis with myocardial injury, myocardial edema and pericardial effusion (**Figures 2A–D**). We initiated corticosteroid treatment again, in combination with prophylactic aspirin for antiplatelet aggregation and low molecular weight heparin therapy. As shown in **Figure 1B**, the patient’s clinical indicators showed improvement.

At a follow-up after 5 months, CMR revealed complete resolution of pericardial effusion and myocardial edema (**Figures 2E–H**). Subsequent laboratory tests showed Hb levels were normal, Coombs test was negative, and cardiac biomarkers including TnI, hs-TnT, CK-MB, and NT-Pro BNP returned to normal. The aPL remained consistently positive. The patient continued to take a low dose of corticosteroids and aspirin. During our close follow-up, the patient remained in good health.

## 3 Literature review

**Figure 3:**
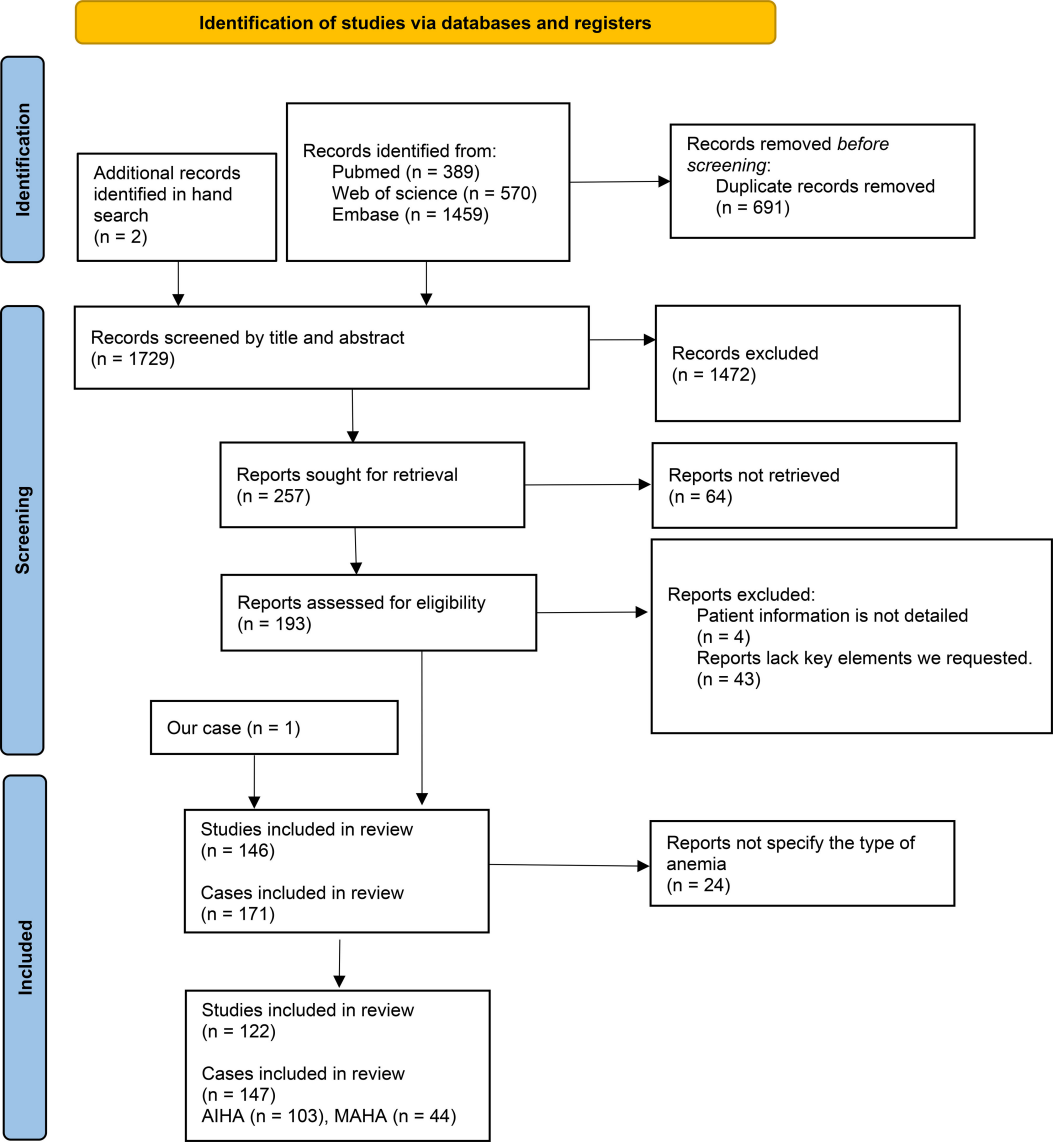
PRISMA flow diagram for the study.

We conducted a comprehensive search of the literatures related to “antiphospholipid syndrome” and “hemolytic anemia” published before March 13, 2025 in PubMed, Web of Science and Embase databases. We collected cases with complete clinical data and a confirmed diagnosis of APS accompanied by hemolytic anemia, while excluding those with congenital hereditary hemolytic disorders and studies that did not specify the presence of hemolytic anemia at the individual patient level. The literature screening process is detailed in **Figure 3**. Of these studies, two were retrospective analyses and the others were case reports. In the included literature, we further screened for cases where cardiac complications were clearly identified. Data extracted included the age of onset, gender, cardiac clinical manifestations, classification of hemolytic anemia, and laboratory test results for aPL (**Supplementary Table 1**).

The initial literature search yielded 146 results, including our case, resulting in a total of 171 patients after the removal of duplicates. To investigate the likelihood of cardiac complications in patients with APS associated with different types of anemia, the anemias were ultimately categorized into autoimmune hemolytic anemia (AIHA) (n = 103) and microangiopathic hemolytic anemia (MAHA) (n = 44), with 24 cases having an unspecified type of anemia. Specific information is provided in **Supplementary Table 2**. Manual screening identified 35 patients with APS associated with hemolytic anemia and cardiac disease, along with our reported case, were included in **Supplementary Table 1** for statistical analysis.

All 147 cases of APS with definite hemolytic anemia, we found that MAHA is associated with a significantly higher incidence of cardiac complications compared to AIHA (36.4% vs. 18.4%, p = 0.020) (see **Table 1**).

**Table 1:** Differences in cardiac complications among antiphospholipid syndrome patients with various type of hemolytic anemia.

Cardiac complications	AIHA (n, %)	MAHA (n, %)	P*
Yes	19 (18.4)	16 (36.4)	0.020
No	84 (81.6)	28 (63.6)	

*Chi-square test. p<0.05.

Among 35 APS patients who had hemolytic anemia and cardiac disease, the median age of onset was 32 years (ranging from 7 to 72 years, **Supplementary Table 1**). 10 patients were under the age of 18, accounting for 28.6% of the total. Males were 25.7% (9/35), while females accounted for 74.3% (26/35). Among the 35 APS patients, 13 cases reported the time window from hemolytic anemia to the onset of cardiac complications, with a median interval of 3 months (ranging from 5 days to 2 years). Among these 13 cases, 12 (92.3%) experienced cardiac complications within one year. The median age of these 13 patients was 14 years (with a range of 7 to 54 years), including 7 patients (53.8%) under the age of 18.

Notably, among 35 APS patients with both hemolytic anemia and cardiac disease, myocardial complications accounted for 62.9% (22/35), valvular disease for 25.7% (9/35), and intracardiac thrombosis for 14.3% (5/35). Among the myocardial complications, there were 8 cases of myocardial infarction, 6 cases of heart failure, 3 cases of cardiomyopathy, and 3 cases of myocarditis.

## 4 Discussion

We report a child presenting with AIHA, myocarditis, and positive aPLs, who was diagnosed with suspected APS. The patient’s ECG did not show ST segment changes, and both echocardiography and CTA did not reveal any thrombi. However, CMR indicated myocarditis with myocardial injury, myocardial edema, and pericardial effusion. Given the presence of prolonged APTT and multiple aPL, we strongly suspected APS. After initiating treatment with corticosteroids and prophylactic low molecular weight heparin for anticoagulation, the patient’s abnormal parameters improved, and subsequent follow-up did not reveal any thrombotic manifestations. Notably, this case is particularly interesting because, based on the limited diagnostic findings available, the cardiac involvement in this APS patient likely differs from typical arterial thrombotic features (17) associated with APS. It is more likely to be a non-thrombotic form of myocardial disease. However, we acknowledge that since no biopsy was performed, we cannot definitively rule out the possibility of microangiopathic lesions. To our knowledge, this is the first reported case of myocarditis in a child with primary APS. This finding broadens the understanding of multi-organ manifestations in APS and highlights myocarditis as a potential rare yet important clinical phenotype.

APS is known for its thrombotic clinical manifestations, and hemolytic anemia, considered a non-thrombotic manifestation of APS, may influence cardiac complications through several mechanisms, including the onset of myocardial ischemia, an increase in cardiac workload, the activation of inflammatory responses, and the hypercoagulable state (10, 18–23). From our analysis of 171 APS patients with hemolytic anemia, we found that 4.7% (8/171) experienced myocardial infarction, which is significantly higher than the reported incidence of first-onset myocardial infarction in the general APS patient cohorts studied by Hui Shi (1.2%) (24) and Cervera (2.8%) (25). Additionally, 1.8% (3/171) experienced myocarditis, also exceeding the reported incidence of myocarditis in the general APS patient cohort of the Yuan Zhao’s study (0.9%) (26). This phenomenon suggests that hemolytic anemia may be associated with an increased risk of cardiac complications in APS patients. Furthermore, in our analysis, MAHA is more likely to be associated with cardiac complications compared to AIHA (P = 0.020) (**Table 1**). This increased risk may be related to the fact that nearly half of these 35 cases evolved into CAPS, where the underlying mechanism includes extensive microvascular and macrovascular changes due to intravascular thrombosis (27). This situation is more likely to present clinical manifestations of both hemolytic anemia and cardiac complications compared to classical APS. Additionally, MAHA often leads to mechanical damage to erythrocytes from thrombus formation in micro-vessels. These microthrombi can not only destroy erythrocytes but may also occlude cardiac micro-vessels, potentially resulting in myocardial ischemia or infarction (28, 29). However, it is important to note that the articles included in our review are primarily case reports. Case reports often describe more severe conditions, which may lead to publication bias; consequently, the absolute frequencies presented here could overestimate the actual prevalence of complications in the general population.

In the analysis of the 13 cases that reported the time window from hemolytic anemia to the onset of cardiac complications (**Supplementary Table 1**), we observed a wide variation in the time window, ranging from several days to several years, with a median interval of 3 months. Notably, 92.3% (12 cases) experienced the onset of cardiac complications within one year, with some patients even undergoing anticoagulant therapy during this period. Therefore, for APS patients with concomitant hemolytic anemia, clinicians need to be aware of the potential for cardiac complications. Notably, the majority of these cases were children (53.8%), highlighting the need for special attention to the characteristics of disease progression in pediatric patients.

As shown in **Supplementary Table 1**, among the 35 cases of hemolytic anemia associated with cardiac complications, 28.6% were children under the age of 18, with nearly half of these pediatric patients (4/10) presenting with hemolytic anemia as the initial symptom. Interestingly, similar to the cases we reported, 12 cases exhibiting hemolytic anemia as the initial symptom were identified in **Supplementary Table 1**. These data add to the evidence that Ped-APS patients may present with non-thrombotic manifestations. Currently, there are no independent classification standards for Ped-APS, and classification criteria still relies on adult criteria (30). However, the clinical presentations of APS in children are different from those in adults, as many traditional thrombotic risk factors, such as smoking and hypertension, are rare in pediatric populations (4). In contrast, non-thrombotic manifestations, such as hematological abnormalities, microvascular lesions, and neurological symptoms, are more prevalent in Ped-APS than in adults, and their importance cannot be ignored, which may be an important marker of early disease (4, 31, 32).

Regarding the therapy of APS, our patient received corticosteroids as the first-line treatment for AIHA in the early stage of the disease (33). Laboratory tests revealed triple-positive aPL profiles, accompanied by prolonged APTT, indicating a high risk of APS (34). Although no thrombotic events were observed and the patient did not meet the classification criteria for APS, we administered anticoagulant therapy (prophylactic low molecular weight heparin). Once the patient’s Hb level returned to normal, we discontinued corticosteroids. However, shortly after drug withdrawal, the patient developed asymptomatic myocarditis, and it was highly suspected to be APS. While the primary treatment for APS usually does not focus primarily on corticosteroids, the presence of myocarditis as a significant clinical manifestation prompted us to reinitiate corticosteroids, along with prophylactic low molecular weight heparin for anticoagulation (35). Additionally, aspirin was included for antiplatelet therapy to prevent thrombosis. The patient responded well to this treatment plan. From this case, it is evident that in managing Ped-APS, it is crucial to emphasize not only the necessary anti-thrombotic therapy but also the treatment of non-thrombotic clinical manifestations. Their potential application in pediatric patients deserves further exploration (36). Therefore, based on the existing evidence, we advocate that future Ped-APS treatment guidelines should thoroughly address treatment regimens specifically targeting non-thrombotic symptoms.

## 5 Conclusion

In conclusion, APS patients presenting with hemolytic anemia, especially MAHA, may be at an elevated risk for complications such as myocardial infarction and myocarditis. Therefore, it may be beneficial to maintain heightened vigilance for cardiac complications in APS patients, particularly those with concurrent hemolytic anemia. Prompt cardiac evaluation is advised at initial presentation and during subsequent follow-up. Furthermore, although Ped-APS is rare, it is essential to enhance awareness of this condition in children, particularly regarding non-thrombotic manifestations such as hemolytic anemia and myocarditis, which may even present as initial symptoms of APS. It may be beneficial to give more attention to non-thrombotic manifestations, particularly when developing criteria for children.

## Data Availability

The original contributions presented in the study are included in the article/**Supplementary Material**. Further inquiries can be directed to the corresponding author.
