# Supplementary material for: Don’t neglect the non-thrombotic manifestations of antiphospholipid syndrome in children – autoimmune hemolytic anemia and myocarditis: a case report and literature review
Source: Front Immunol. 2026 Jan 30;17:1724748. doi: 10.3389/fimmu.2026.1724748 (PMC12901497; doi:10.3389/fimmu.2026.1724748)
Supplement: Supplementary file 1 [file DataSheet1.pdf]

Supplementary Material

Supplementary Table 1 Cases of antiphospholipid syndrome associated with hemolytic anemia and cardiac complications.

No.	Year	Age (years)	Gender	Diagnosis	Cardiac manifestations	Type of hemolytic anemia	Time window [b]	aPL test			
								aCL (IgG/IgM)	anti-β_2GPI (IgG/IgM)	LA	aPL positive duration
1(1)	2019	52	F	CAPS, SLE, TMA	Acute congestive heart failure, severe cardiomyopathy, severe mitral and aortic regurgitation	MAHA		NA	NA	NA	NA
2(2)	1998	37	F	CAPS, SLE	Secondary systemic amyloidosis with perivascular deposit of amyloid (AA type) in the heart	AIHA [a]		+	NA	+	NA
3(3)	1999	67	F	CAPS	Acutely congestive heart failure, cardiomegaly	MAHA [a]		+	NA	+	NA
4(4)	2013	32	F	CAPS, new-onset SLE, TMA	Cardiomyopathy, valvular heart disease	MAHA [a]		-	-	+	NA
5(5)	2014	36	F	CAPS	NSTEMI	MAHA		-	-	+	NA
6(6)	2019	45	F	CAPS, swine flu (HINI)	Ball like vegetations on mitral and aortic valve	AIHA		NA	NA	+	NA
7(7)	2004	22	F	PAPS	Mitral valve thickening	MAHA		+	NA	+	once
8(7)	2004	18	F	PAPS	Mitral valve thickening	MAHA		+	NA	+	once
9(8)	2010	72	F	PAPS	Pulmonary hypertension	MAHA [a]		+	+	+	NA
10(9)	2024	44	F	APS, SCAD	SCAD, Congestive heart failure, NSTEMI, new ST-segment elevation	MAHA		NA	NA	NA	NA
11(10)	2015	54	M	PAPS	Organised vegetations over the mitral valve	cold AIHA [a]		+	NA	NA	6 weeks
12(11)	2012	9	F	SLE, SAPS, NF1	Aortic valvulopathy	AIHA		+	NA	+	once
13(12)	2023	72	M	CAPS	Pericardial effusion with tamponade physiology	MAHA		+	+	NA	NA
14(13)	2019	48	F	APS, SLE	cardiac mass	AIHA		+	NA	NA	once

15(14)	2024	32	M	SLE, secondary CAPS	Myocarditis with associated microvascular occlusion (MVO)	AIHA [a]		NA	NA	NA	NA
16(15)	2025	26	F	APS, SLE, ES, BCS	A thrombus in the right atrium	AIHA		-	-	+	once
17(16)	2023	49	M	CAPS	Multiple large masses attached to aortic valve and mitral valve	MAHA		+	NA	+	NA
18(17)	2023	52	F	CAPS	Cardiomegaly, cardiogenic pulmonary edema.<br>Troponins trended upwards	MAHA		NA	NA	NA	NA
19(18)	2018	19	F	CAPS	NSTEMI	MAHA		-	NA	+	NA
20(19)	2023	7	M	CAPS, SLE	Diffuse ST inversion and a prolonged QTc interval, pericardial effusion	AIHA		+	NA	NA	NA
21(20)	2012	22	F	APS, SLE	Biventricular thrombus, endomyocardial fibrosis, mild pulmonary hypertension, mild pericardial effusion	AIHA [a]		+	NA	NA	once
22(21)	2010	33	F	PAPS with lupus-like disease	Mobile mitral and aortic valve vegetations,<br>severe mitral regurgitation, infective endocarditis,<br>congestive heart failure, cardiomegaly,<br>myocardial ischemia	AIHA		+	NA	+	>12 weeks
23≠(13)	2019	13	F	APS, SLE	A cardiac mass, which was compatible with myxoma, pericardial effusion	AIHA	1 year	+	NA	+	once
24≠(22)	2024	8	F	APS	MI,<br>a thrombus-like clot in the left auricle,<br>trace pericardial effusion	AIHA [a]	2 years	+	+	+	NA
25≠(23)	2000	7	M	APS, HUS	Severe dilated cardiomyopathy, MI	MAHA	7 months	+	NA	+	8 weeks
26≠(24)	2002	51	F	APS	A 3-cm intracardiac thrombus	AIHA [a]	5days	+	+	+	NA
27≠(25)	2004	32	M	CAPS	Acute coronary syndrome (angina), mild ischaemia	MAHA	5days	+	NA	+	NA
28≠(26)	2012	7	F	CAPS, TMA	Hypokinesia of the apical segment of heart,	MAHA [a]	5days	+	NA	+	NA

					suggestive of coronary involvement						
29[≠](27)	1996	28	F	APS, SLE	MI	AIHA	10 months	+	NA	+	>12 weeks
30[≠](28)	2011	11	F	APS, SLE, ES	Congestive cardiac failure	AIHA [a]	4 months	NA	NA	NA	NA
31[≠](29)	2021	54	F	APS with HIV(-) CM	Heart failure	AIHA	1 year	+	+	+	NA
32[≠](30)	2023	31	F	SLE, SAPS, ES	4 intracardiac thrombi were found in the right atrium	AIHA [a]	1 month	+	-	NA	once
33[≠](31)	2019	14	F	CAPS, SLE	Acute MI, hypofunction of the right ventricle, myocarditis	AIHA	3 months	+	+	+	NA
34[≠](32)	2012	40	M	CAPS	inferior and septal MI	MAHA	2 months	+	NA	+	NA
35[≠] Our case	2024	13	M	APS	Myocarditis, pericardial effusion	AIHA [a]	2 months	+	+	+	NA

F: female, M: male, APS: Antiphospholipid Syndrome, CAPS: Catastrophic Antiphospholipid Syndrome, SLE: Systemic Lupus Erythematosus, TMA: Thrombotic Microangiopathy, PAPS: Primary Antiphospholipid Syndrome, SCAD: Spontaneous Coronary Artery Dissection, SAPS: Secondary Antiphospholipid Syndrome, NF1: Neurofibromatosis type 1, ES: Evans Syndrome, BCS: Budd-Chiari Syndrome, HUS: Hemolytic Uremic Syndrome, HIV(-) CM: HIV negative cryptococcal meningitis, MI: Myocardial infarction, NSTEMI: Non-ST segment myocardial infarction, AIHA: Autoimmune Hemolytic Anemia, MAHA: Microangiopathic Hemolytic Anemia, aPL: antiphospholipid antibodies, aCL: anticardiolipin antibody, anti-β2GPI: anti-β2 glycoprotein 1 antibody, LA: lupus anticoagulant, NA: Not Applicable

13 APS cases[≠] developed hemolytic anemia first, followed by cardiac issues some time later.

[a] This indicates that APS patients present with hemolytic anemia as the initial symptom.

[b] This represents the time interval from the onset of hemolytic anemia to the development of cardiac involvement.

Supplementary Table 2 Cases of antiphospholipid syndrome with concomitant hemolytic anemia.

AIHA				MAHA		Unknown	
Cardiac disease	No cardiac disease			Cardiac disease	No cardiac disease	Cardiac disease	No cardiac disease
Derksen *et al.* (27)	Li *et al.* (33)	Khalifa *et al.* (34)	Zhang *et al.* (35)	Cerveny *et al.* (3)	Kosaka *et al.* (36)	Ketari Jamoussi *et al.* (37)	Huang *et al.* (38)
Asherson *et al.* (2)	Murphy *et al.* (39)	Musso *et al.* (40)	Corredor-Orlandelli *et al.* (41)	do Sameiro Faria *et al.* (23)	Shiari *et al.* (42)	Shumilova *et al.* (43)	Meyers *et al.* (44)
Latagliata *et al.* (24)	Durukan *et al.* (45)	Font *et al.* (46)	Suzuki *et al.* (47)	Amoura *et al.* (7)	Wig *et al.* (48)	Bowden *et al.* (49)	Gerner *et al.* (50)
Patra *et al.* (28)	Ravelli *et al.* (51)	Gurudu *et al.* (52)	Lijia *et al.* (53)	Espinosa *et al.* (25)	Ho *et al.* (54)	Noda *et al.* (55)	Quéméneur *et al.* (56)
Ediriweera *et al.* (10)	De-Leon-Bojorge *et al.* (57)	Ibrahim *et al.* (58)	Matsuura *et al.* (59)	Ishigaki *et al.* (8)	Berry *et al.* (60)		Sakhel *et al.* (61)
Madkaiker (6)	Funauchi *et al.* (62)	Sherer *et al.* (63)	Takahashi *et al.* (64)	Prasad *et al.* (26)	Korotchaeva *et al.* (65)		Zeller *et al.* (66)
Zhao *et al.* (29)	Athanazio *et al.* (67)	Alba *et al.* (68)	Rymarz *et al.* (69)	Vieregge *et al.* (4)	Basbayraktar *et al.* (70)		Le Guilloux *et al.* (71)
Zheng *et al.* (22)	Modrykamien *et al.* (72)	Fernando *et al.* (73)	Pfeiff *et al.* (74)	Strakhan *et al.* (5)	Meglic *et al.* (75)		Grossman *et al.* (76)
Akyüz *et al.* (11)	Sáez-de-Ocariz *et al.* (77)	Rückert *et al.* (78)	Krysiak *et al.* (79)	Tulai *et al.* (1)	Guevara-Rodriguez *et al.* (80)		Kew *et al.* (81)
León-Jiménez *et*	Yong *et al.* (82)	Inoue *et al.* (83)	Aburahma (84)	Perez *et al.* (9)	Bhadauria *et al.*		Tian *et al.* (86)

al. (30)					(85)		
Giráldez *et al.* (13)	Torres-Jimenez *et al.* (87)	Gonzalez-Nieto *et al.* (88)	Hadler *et al.* (89)	Alonso *et al.* (18)	Horikoshi *et al.* (90)		Horita *et al.* (91)
Shin *et al.* (31)	Haselboeck *et al.* (92)	Lopetegui-Lia *et al.* (93)	Mekala *et al.* (94)	Fernandez *et al.* (17)	Kniaz *et al.* (95)		Barbour *et al.* (96)
Wong *et al.* (19)	Diószegi *et al.* (97)	Scaramucci *et al.* (98)	Núñez-Álvarez *et al.* (99)	Lewien *et al.* (12)	Nagayama *et al.* (100)		Yousuf *et al.* (101)
Ganes *et al.* (14)	Dhanawat *et al.* (102)	Del Proposto *et al.* (103)	Abdelhalim *et al.* (104)	Tseng (16)	Asherson *et al.* (105)		Espino *et al.* (106)
Porcu *et al.* (15)	Siu *et al.* (107)	Gong *et al.* (108)	Lu *et al.* (109)	Park *et al.* (32)	Sakaguchi *et al.* (110)		Sivasailam *et al.* (111)
Yee *et al.* (21)	Arvieux *et al.* (112)	Tkachenko *et al.* (113)	Papadopoulos *et al.* (114)		Tanariyakul *et al.* (115)		Suenaga *et al.* (116)
Gonçalves *et al.* (20)	Font *et al.* (117)	Patel *et al.* (118)	Vandenberghe *et al.* (119)		Díaz-Cremades *et al.* (120)		Koenig *et al.* (121)
	Fonseca *et al.* (122)	Gelman *et al.* (123)	Raupov *et al.* (124)		Mutter *et al.* (125)		Suzumori *et al.* (126)
	Ruffatti *et al.* (127)	Nauseef *et al.* (128)	Paydas *et al.* (129)		Kolasinski (130)		Chang *et al.* (131)
	Sopeña *et al.* (132)	Veskitkul *et al.* (133)	Baya *et al.* (134)		de Holanda *et al.* (135)		Vassia *et al.* (136)
	Breccia *et al.* (137)	Nauseef *et al.* (138)	Demir *et al.* (139)		Rovere-Querini *et al.* (140)		

					Stanescu *et al.* (141)		
					Gallant *et al.* (142)		
					Oulego-Erroz *et al.* (143)		
					Hanai *et al.* (144)		
					Lazurova *et al.* (145)		
					Nawata *et al.* (146)		

1. Tulai IM, Penciu OM, Raut R, and Rudinskaya A. Catastrophic Antiphospholipid Syndrome Presenting as Congestive Heart Failure in a Patient with Thrombotic Microangiopathy. *Tex Heart Inst J.* (2019) 46: 48-52. doi: 10.14503/thij-17-6472.
2. Asherson RA, Cervera R, Piette JC, Font J, Lie JT, Burcoglu A, et al. Catastrophic antiphospholipid syndrome. Clinical and laboratory features of 50 patients. *Medicine (Baltimore).* (1998) 77: 195-207. doi: 10.1097/00005792-199805000-00005.
3. Cerveny KC, and Sawitzke AD. Relapsing catastrophic antiphospholipid antibody syndrome: a mimic for thrombotic thrombocytopenic purpura? *Lupus.* (1999) 8: 477-481. doi: 10.1177/096120339900800613.
4. Vieregge GB, Harrington TJ, Andrews DM, Carpintero MF, Green DF, and Nayer A. Catastrophic antiphospholipid syndrome with severe acute thrombotic microangiopathy and hemorrhagic complications. *Case Rep Med.* (2013) 2013: 915309. doi: 10.1155/2013/915309.
5. Strakhan M, Hurtado-Sbordoni M, Galeas N, Bakirhan K, Alexis K, and Elrafei T. 36-year-old female with catastrophic antiphospholipid syndrome treated with eculizumab: a case report and review of literature. *Case Rep Hematol.* (2014) 2014: 704371. doi: 10.1155/2014/704371.
6. Madkaiker S. Catastrophic Antiphospholid Syndrome - An Unusual Case Report. *Indian J Crit Care Med.* (2019) 23: 276-280. doi: 10.5005/jp-journals-10071-23180.
7. Amoura Z, Costedoat-Chalumeau N, Veyradier A, Wolf M, Ghillani-Dalbin P, Cacoub P, et al. Thrombotic thrombocytopenic purpura with severe ADAMTS-13 deficiency in two patients with primary antiphospholipid syndrome. *Arthritis Rheum.* (2004) 50: 3260-3264. doi: 10.1002/art.20551.
8. Ishigaki K, Takizawa Y, Maruyama J, and Setoguchi K. Pulmonary thrombotic microangiopathic hemolytic anemia treated successfully with anticoagulant monotherapy. *Intern Med.* (2010) 49: 1217-1220. doi: 10.2169/internalmedicine.49.3315.
9. Perez E, Guevara N, Smith J, and Velasquez R. Altered Mental Status in the Setting of Thrombotic Thrombocytopenic Purpura (TTP) and Spontaneous Coronary Artery Dissection (SCAD): A Case Report and Literature Review. *Cureus.* (2024) 16: e54642. doi: 10.7759/cureus.54642.
10. Ediriweera HM, Gayani GG, Pathirana KD, Weerarathna TP, and Mohideen MR. Antiphospholipid syndrome in a man presenting with cold autoimmune haemolytic anaemia. *Ceylon Med J.* (2015) 60: 71-72. doi: 10.4038/cmj.v60i2.8156.
11. Akyüz SG, Çaltik A, Bülbül M, Erdogan Ö, Renda R, and Demircin G. An unusual pediatric case with neurofibromatosis and systemic lupus erythematosus. *Rheumatology International.* (2012) 32: 2345-2347. doi: 10.1007/s00296-011-1966-z.
12. Lewien P, McCreary D, Ridenour L, Singarajah CU, and Garcia Orr R. A kidney conundrum: antiphospholipid syndrome masquerading as scleroderma renal crisis. *Chest.* (2023) 164: A2754-A2755. doi: 10.1016/j.chest.2023.07.1817.

13. Giráldez CR, Aulestia NP, Feijoo MLV, and De La Fuente JLM. Intracardiac tumor vs thrombus in patients with antiphospholipid syndrome: Two case reports. *Lupus Science and Medicine.* (2019) 6: A105. doi: 10.1136/lupus-2019-lsm.145.
14. Ganes A, Sethwala A, Hengel C, and Yao J. Unveiling the Heart of Lupus: A Rare Presentation of SLE as First Episode Myocarditis With Catastrophic Antiphospholipid Syndrome. *Heart Lung and Circulation.* (2024) 33: S375. doi: 10.1016/j.hlc.2024.06.540.
15. Porcu C, Merkel N, and Fusi-Schimdhauser T. Budd-chiari syndrome as an initial manifestation of incomplete systemic lupus erythematosus. *European Journal of Case Reports in Internal Medicine.* (2025) 12. doi: 10.12890/2024_005015.
16. Tseng C. Catastrophic antiphospholipid syndrome (CAPS) diagnosed post-mortem: lessons learned. *Chest.* (2023) 164: A5682. doi: 10.1016/j.chest.2023.07.3667.
17. Fernandez JC, Porto J, Ortiz C, Melendez Gonzalez JE, Zapata DI, and Arshed SK. Surviving catastrophe: a case of antiphospholipid syndrome-induced multiorgan failure. *Chest.* (2023) 164: A5655-A5656. doi: 10.1016/j.chest.2023.07.3650.
18. Alonso JV, del Pozo FJF, Álvarez MV, Pedraza J, Aguayo MA, and Sanchez A. Catastrophic antiphospholipid antibody syndrome presenting as acute vascular occlusion in a young female patient. *Reumatologia Clinica.* (2018) 14: 49-52. doi: 10.1016/j.reuma.2016.09.002.
19. Wong CK, Fang LC, Hung WL, and Wu YH. Acrocyanosis and retiform purpura as the first manifestation of catastrophic antiphospholipid syndrome in a child: A case report. *Dermatologica Sinica.* (2023) 41: 54-55. doi: 10.4103/ds.DS-D-22-00086.
20. Gonçalves LFG, Souto FMS, Faro FN, Oliveira JLM, Barreto-Filho JAS, and Sousa ACS. Biventricular thrombus and endomyocardial fibrosis in antiphospholipid syndrome. *Arquivos Brasileiros de Cardiologia.* (2012) 99: e162-e165. doi: 10.1590/S0066-782X2012001400017.
21. Yee DZ, Teng GG, Lim AY, Low AF, and Vasoo S. The autoimmunity conundrum: clotting or inflammation. *Int J Rheum Dis.* (2010) 13: e62-66. doi: 10.1111/j.1756-185X.2010.01534.x.
22. Zheng J, Wei ZY, Lin SC, Wang Y, and Fang X. Antiphospholipid syndrome onset with hemolytic anemia and accompanied cardiocerebral events: a case report. *Front Pediatr.* (2024) 12: 1370285. doi: 10.3389/fped.2024.1370285.
23. do Sameiro Faria M, Mota C, Barbot J, Alvares S, Jardim H, Vilarinho A, et al. Haemolytic uraemic syndrome, cardiomyopathy, cutaneous vasculopathy and anti-phospholipid activity. *Nephrol Dial Transplant.* (2000) 15: 1891-1892. doi: 10.1093/ndt/15.11.1891.
24. Latagliata R, Celesti F, Bongarzoni V, Di Nucci G, Torromeo C, Morano SG, et al. Intracardiac thrombus in a patient with autoimmune hemolytic anemia leading to a diagnosis of antiphospholipid syndrome. *Acta Haematol.* (2002) 107: 170-172. doi: 10.1159/000057635.
25. Espinosa G, Bucciarelli S, Cervera R, Lozano M, Reverter JC, de la Red G, et al. Thrombotic microangiopathic haemolytic anaemia and antiphospholipid antibodies. *Ann Rheum Dis.* (2004) 63: 730-736. doi: 10.1136/ard.2003.007245.
26. Prasad N, Bhadauria D, Agarwal N, Gupta A, Gupta P, Jain M, et al. Catastrophic antiphospholipid antibody syndrome in a child with thrombotic microangiopathy. *Indian J Nephrol.* (2012) 22: 310-313. doi: 10.4103/0971-4065.101266.
27. Derksen RH, Gmelig-Meijling FH, and de Groot PG. Primary antiphospholipid syndrome evolving into systemic lupus erythematosus. *Lupus.* (1996) 5: 77-80. doi: 10.1177/096120339600500115.
28. Patra S, Krishnamurthy S, Seth A, Beri S, and Aneja S. Bilateral optic neuritis in pediatric systemic lupus erythematosus with antiphospholipid antibody syndrome. *Indian J Pediatr.* (2011) 78: 234-236. doi: 10.1007/s12098-010-0228-5.

29. Zhao J, Wu X, Huang Z, and Zhang J. A case of HIV negative cryptococcal meningitis with antiphospholipid syndrome. *Zhong Nan Da Xue Xue Bao Yi Xue Ban.* (2021) 46: 438-443. doi: 10.11817/j.issn.1672-7347.2021.200471.

30. León-Jiménez FE, and Juarez-Lloclla JP. Síndrome antifosfolípido y tumores cardiacos. *Anales de la Facultad de Medicina.* (2023) 84: 97-100. doi: 10.15381/anales.v84i1.24174.

31. Shin JS, and Kim KN. Catastrophic Antiphospholipid Syndrome Associated with Systemic Lupus Erythematosus Successfully Treated with Rituximab: A Case Report. *Journal of Rheumatic Diseases.* (2019) 26: 74-78. doi: 10.4078/jrd.2019.26.1.74.

32. Park HC, Yoon HB, Lee TW, Jung JY, Chin HJ, Kim YS, et al. Recurrent thrombotic events after catastrophic antiphopholipid syndrome. *Korean Journal of Internal Medicine.* (2012) 27: 232-234. doi: 10.3904/kjim.2012.27.2.232.

33. Li HY, Yang WS, Tai TY, and Chuang LM. A diabetic subject with MELAS and antiphospholipid syndrome. *Diabetes care.* (2003) 26: 2218-2219. doi: 10.2337/diacare.26.7.2218.

34. Khalifa M, Ghannouchi N, Kaabia N, BenJazia E, Hachfi W, Krifa A, et al. Primary antiphospholipid syndrome and Evan's syndrome: 2 case reports. *Acta Clin Belg.* (2009) 64: 65-67. doi: 10.1179/acb.2009.012.

35. Zhang D, Sun F, and Ye S. Successful treatment of sirolimus in a Chinese patient with refractory LN and APS: a case report. *Ther Adv Musculoskelet Dis.* (2022) 14: 1759720x221079253. doi: 10.1177/1759720x221079253.

36. Kosaka M, Takahashi N, Saitoh H, Masai R, Ito M, Sato R, et al. Thrombotic thrombocytopenic purpura with severe ADAMTS-13 deficiency in a patient with antiphospholipid antibodies and Charcot-Marie-Tooth disease. *Intern Med.* (2011) 50: 487-493. doi: 10.2169/internalmedicine.50.4300.

37. Ketari Jamoussi S, Zaghdoudi I, Ben Dhaou B, Kochbati S, Mir K, Ben Ali Z, et al. Catastrophic antiphospholipid syndrome and rituximab: a new report. *Tunis Med.* (2009) 87: 699-702. doi:

38. Huang JJ, Chen MW, Sung JM, Lan RR, Wang MC, and Chen FF. Postpartum haemolytic uraemic syndrome associated with antiphospholipid antibody. *Nephrol Dial Transplant.* (1998) 13: 182-186. doi: 10.1093/ndt/13.1.182.

39. Murphy PT, and Rao P. Central retinal venous occlusion with co-existent thrombotic thrombocytopenic purpura and antiphospholipid syndrome. *British Journal of Ophthalmology.* (2003) 87: 658-659. doi: 10.1136/bjo.87.5.658.

40. Musso M, Porretto F, Crescimanno A, Bondi F, Polizzi V, Scalone R, et al. Autologous peripheral blood stem and progenitor (CD34+) cell transplantation for systemic lupus erythematosus complicated by Evans syndrome. *Lupus.* (1998) 7: 492-494. doi: 10.1191/096120398678920424.

41. Corredor-Orlandelli D, Arévalo-Romero A, Reyes C, and Arango D. Massive Right Chylothorax Secondary to a Severe Systemic Lupus Erythematosus Flare With Secondary Evans Syndrome: A Case Report and Literature Review. *Clin Med Insights Case Rep.* (2023) 16: 11795476231186735. doi: 10.1177/11795476231186735.

42. Shiari R, Parvaneh VJ, Dalirani R, Farivar S, and Shiva MR. Atypical hemolytic-uremic syndrome associated with antiphospholipid antibodies and antiphospholipid syndrome; A novel presentation. *Pediatric Rheumatology.* (2014) 12. doi:

43. Shumilova A, Reshetnyak T, Cheldieva F, Cherkasova M, and Lila A. Case report of a systemic lupus erythematosus and antiphospholipid syndrome patient with an infiltrative tuberculosis and melanoma: Features of therapeutic approaches. *Lupus Science and Medicine.* (2020) 7: A108-A109. doi: 10.1136/lupus-2020-eurolupus.203.

44. Meyers KE, Pfieffer S, Lu T, and Kaplan BS. Genitourinary complications of systemic lupus erythematosus. *Pediatr Nephrol.* (2000) 14: 416-421. doi: 10.1007/s004670050786.
45. Durukan AH, Akar Y, Bayraktar MZ, Dinc A, and Sahin OF. Combined retinal artery and vein occlusion in a patient with systemic lupus erythematosus and antiphospholipid syndrome. *Canadian Journal of Ophthalmology.* (2005) 40: 87-89. doi: 10.1016/S0008-4182(05)80126-7.
46. Font J, Jiménez S, Cervera R, García-Carrasco M, Ramos-Casals M, Campdelacreu J, et al. Splenectomy for refractory Evans' syndrome associated with antiphospholipid antibodies: report of two cases. *Ann Rheum Dis.* (2000) 59: 920-923. doi: 10.1136/ard.59.11.920.
47. Suzuki E, Kanno T, Saito Y, and Shimbo T. Systemic Lupus Erythematosus and Antiphospholipid Syndrome Accompanied by Mixed-Type Autoimmune Hemolytic Anemia. *Case Rep Rheumatol.* (2023) 2023: 4963196. doi: 10.1155/2023/4963196.
48. Wig S, Chan M, Bruce I, and Barnes T. Successful management of a case of relapsing and refractory catastrophic antiphospholipid antibody syndrome with eculizumab, a complement 5A inhibitor. *Rheumatology (United Kingdom).* (2015) 54: i58. doi: 10.1093/rheumatology/kev088.024.
49. Bowden A, Walsh R, Murphy R, and Sexton D. A case of Libman–Sacks endocarditis in a patient with systemic lupus erythematosus and antiphospholipid syndrome. *European Heart Journal - Case Reports.* (2024) 8. doi: 10.1093/ehjcr/ytae503.
50. Gerner P, Heldmann M, Borusiak P, Bures V, and Wirth S. Adrenal failure followed by status epilepticus and hemolytic anemia in primary antiphospholipid syndrome. *Thromb J.* (2005) 3: 6. doi: 10.1186/1477-9560-3-6.
51. Ravelli A, and Martini A. Antiphospholipid syndrome. *Pediatr Clin North Am.* (2005) 52: 469-491, vi. doi: 10.1016/j.pcl.2005.01.001.
52. Gurudu SR, Mittal SK, Shaber M, Gamboa E, Michael S, and Sigal LH. Autoimmune hepatitis associated with autoimmune hemolytic anemia and anticardiolipin antibody syndrome. *Dig Dis Sci.* (2000) 45: 1878-1880. doi: 10.1023/a:1005501421242.
53. Lijia S, Shuo Z, Shengchun F, and Yongjun M. "Non-criteria" antiphospholipid syndrome and autoimmune hemolytic anemia: a case report. *Acta Neurol Belg.* (2024) 124: 269-271. doi: 10.1007/s13760-023-02276-0.
54. Ho A, and Koenig S. Diagnostic dilemma: Multiorgan failure due to catastrophic antiphospholipid syndrome in a young woman with six healthy children. *Chest.* (2017) 152: A278. doi: 10.1016/j.chest.2017.08.304.
55. Noda S, Ogura M, Tsutsumi A, Udagawa T, Kamei K, Matsuoka K, et al. Thrombotic microangiopathy due to multiple autoantibodies related to antiphospholipid syndrome. *Pediatric Nephrology.* (2012) 27: 681-685. doi: 10.1007/s00467-011-2085-5.
56. Quéméneur T, Noel LH, Kyndt X, Droz D, Fleury D, Binaut R, et al. Thrombotic microangiopathy in adult Still's disease. *Scand J Rheumatol.* (2005) 34: 399-403. doi: 10.1080/03009740510026689.
57. De-Leon-Bojorge B, Zaltzman-Girsevich S, Ortega-Salgado A, Prieto-Patron A, Córdoba-Córdoba R, and Yamazaki-Nakashimada M. Thrombotic microangiopathy involving the gallbladder as an unusual manifestation of systemic lupus erythematosus and antiphospholipid syndrome: Case report and review of the literature. *World Journal of Gastroenterology.* (2006) 12: 7206-7209. doi: 10.3748/wjg.v12.i44.7206.
58. Ibrahim U, Kedia S, Garcia G, and Atallah JP. Antiphospholipid Syndrome: Multiple Manifestations in a Single Patient-A High Suspicion Is Still Needed. *Case Rep Med.* (2017) 2017: 5797041. doi: 10.1155/2017/5797041.
59. Matsuura Y, Tomita T, Kondo M, Mukai M, and Kataoka H. Severe Thrombocytopenia Secondary to Systemic Lupus Erythematosus With Antiphospholipid Antibodies in a Middle-Aged Woman. *Cureus.* (2024) 16: e62804. doi: 10.7759/cureus.62804.

60. Berry EL, and Iqbal SN. HELLP Syndrome at 17 Weeks Gestation: A Rare and Catastrophic Phenomenon. *J Clin Gynecol Obstet.* (2014) 3: 147-150. doi: 10.14740/jcgo297w.

61. Sakhel K, Usta IM, Hannoun A, Arayssi T, and Nassar AH. Liver infarction in a woman with systemic lupus erythematosus and secondary anti-phospholipid and HELLP syndrome. *Scand J Rheumatol.* (2006) 35: 405-408. doi: 10.1080/03009740600588343.

62. Funauchi M, Yamagata T, Sugiyama M, Ikoma SY, Sakaguchi M, Kinoshita K, et al. A case of antiphospholipid antibody syndrome that manifested in the course of basal cell carcinoma. *Modern Rheumatology.* (2007) 17: 153-155. doi: 10.1007/s10165-006-0550-y.

63. Sherer Y, Dulitzki M, Levy Y, Livneh A, Shoenfeld Y, and Langevitz P. Successful pregnancy outcome in a patient with Gaucher's disease and antiphospholipid syndrome. *Ann Hematol.* (2002) 81: 161-163. doi: 10.1007/s00277-002-0431-1.

64. Takahashi T, Takaoka K, Kwong K, Macapagal S, Tanariyakul M, Wannaphut C, et al. The Successful Management of Stroke in Evans Syndrome by Anticoagulation with Warfarin, Intravenous Immunoglobulin (IVIG), and High-Dose Corticosteroid. *Eur J Case Rep Intern Med.* (2024) 11: 004592. doi: 10.12890/2024_004592.

65. Korotchaeva Y, Kozlovskaya N, Shifman E, and Demyanova K. Successful treatment of postpartum caps by eculizumab. *Nephrology Dialysis Transplantation.* (2018) 33: i385. doi: 10.1093/ndt/gfy104.SP123.

66. Zeller L, Almog Y, Tomer A, Sukenik S, and Abu-Shakra M. Catastrophic thromboses and severe thrombocytopenia during heparin therapy in a patient with anti-phospholipid syndrome. *Clin Rheumatol.* (2006) 25: 426-429. doi: 10.1007/s10067-005-0017-0.

67. Athanazio D, Rocha MC, de Souza e Souza TG, Oliveira e Silva N, Jezler S, and Santiago MB. Chronic catastrophic-like antiphospholipid syndrome: a “smoldering” variant? *Rheumatol Int.* (2009) 30: 123-125. doi: 10.1007/s00296-009-0913-8.

68. Alba P, Karim MY, and Hunt BJ. Mycophenolate mofetil as a treatment for autoimmune haemolytic anaemia in patients with systemic lupus erythematosus and antiphospholipid syndrome. *Lupus.* (2003) 12: 633-635. doi: 10.1191/0961203303lu419cr.

69. Rymarz A, and Niemczyk S. The complex treatment including rituximab in the Management of Catastrophic Antiphospholid Syndrome with renal involvement. *BMC Nephrol.* (2018) 19: 132. doi: 10.1186/s12882-018-0928-z.

70. Basbayraktar B, Al-Jobory OLA, Odimegwu A, Mirembe L, Bellamkonda L, and Afroze T. Thrombotic Thrombocytopenic Purpura with clinical features of Antiphospholipid Syndrome: a challenging diagnosis and management. *Chest.* (2023) 164: A2685-A2686. doi: 10.1016/j.chest.2023.07.1779.

71. Le Guilloux J, Babinet F, Duguay J, Legout A, and Mourand I. Ocular manifestations of thrombotic thrombocytopenic purpura. *Rev Neurol (Paris).* (2009) 165: 486-488. doi: 10.1016/j.neurol.2008.08.002.

72. Modrykamien A, Reddy A, Guzman JA, and Farha S. Massive cerebrovascular infarct due to the catastrophic antiphospholipid syndrome in a patient with idiopathic thrombocytopenic purpura. *Journal of Intensive Care Medicine.* (2009) 24: 269-272. doi: 10.1177/0885066609335756.

73. Fernando MM, and Isenberg DA. Conversion of discoid lupus to antiphospholipid syndrome and SLE. *Nat Clin Pract Rheumatol.* (2008) 4: 106-110. doi: 10.1038/ncprheum0704.

74. Pfeiff R, Constans J, Mayet T, Skopinski S, Barcat D, Guérin V, et al. Catastrophic antiphospholipid syndrome: a new case with favourable outcome. *Revue De Medecine Interne.* (2001) 22: 590-592. doi: 10.1016/s0248-8663(01)00393-9.

75. Meglic A, Grosek S, Benedik-Dolnicar M, and Avcin T. Atypical haemolytic uremic syndrome complicated by microangiopathic antiphospholipid-associated syndrome. *Lupus.* (2008) 17: 842-845. doi: 10.1177/0961203308091634.

76. Grossman A, Green H, Gafter-Gvili A, Zimra Y, Rabizadeh E, and Krause I. Co-existence of paroxysmal nocturnal hemoglobinuria and antiphospholipid syndrome-A role for complement activation. *Leuk Res.* (2010) 34: e251-253. doi: 10.1016/j.leukres.2010.04.022.

77. Sáez-de-Ocariz M, Espinosa-Rosales F, López-Corella E, and de León-Bojorge B. Bullous lesions as a manifestation of systemic lupus erythematosus in two Mexican teenagers. *Pediatric Rheumatology.* (2010) 8. doi: 10.1186/1546-0096-8-19.

78. Rückert A, Glimm H, Lübbert M, and Grüllich C. Successful treatment of life-threatening Evans syndrome due to antiphospholipid antibody syndrome by rituximab-based regimen: a case with long-term follow-up. *Lupus.* (2008) 17: 757-760. doi: 10.1177/0961203307087876.

79. Krysiak R, Kedzia A, and Okopien B. Atypical clinical manifestation of antiphospholipid syndrome. *Polskie Archiwum Medycyny Wewnetrznej-Polish Archives of Internal Medicine.* (2009) 119: 99-102. doi: 10.20452/pamw.618.

80. Guevara-Rodriguez N, Marmanillo-Mendoza G, Castelar J, Ciobanu C, and Fulger I. Unusual presentation of acquired thrombotic thrombocytopenic purpura (TTP) versus catastrophic antiphospholipid syndrome in a patient with Moya-Moya disease, case report, and literature review. *Clinical Case Reports.* (2023) 11. doi: 10.1002/ccr3.7317.

81. Kew GS, Cho J, and Lateef A. Microangiopathic antiphospholipid antibody-associated syndrome in a pregnant lady. *Lupus.* (2017) 26: 435-437. doi: 10.1177/0961203316659548.

82. Yong WH, Kon YC, Rajasoorya C, Goh PNJ, and Howe HS. Bilateral adrenal haemorrhage and evans syndrome as the initial presentation of antiphospholipid syndrome from systemic Lupus erythematosus. *Endocrinologist.* (2010) 20: 66-68. doi: 10.1097/TEN.0b013e3181d54a9d.

83. Inoue D, Togami K, Shimoike N, Tamura R, Imai Y, Kimura T, et al. Early diagnosis and successful treatment of catastrophic antiphospholipid syndrome complicated by multiple organ failure. *Nihon Rinsho Meneki Gakkai Kaishi.* (2010) 33: 24-30. doi: 10.2177/jsci.33.24.

84. Aburahma A. Catastrophic warm autoimmune hemolytic anemia in a patient with primary antiphospholipid syndrome in ICU. *Chest.* (2018) 154: 303A-303A. doi: 10.1016/j.chest.2018.08.278.

85. Bhadauria D, Etta P, Kaul A, and Prasad N. Childhood lupus with microangiopathic antiphospholipid syndrome and pulmonary hemorrhage. *Indian Pediatr.* (2015) 52: 333-334. doi: 10.1007/s13312-015-0634-x.

86. Tian S, Sinclair N, and Shah S. A Case Report of Intravascular Hemolysis and Heme Pigment-Induced Nephropathy Following AngioJet Thrombectomy for Thrombosed DIPS Shunt. *Can J Kidney Health Dis.* (2020) 7: 2054358120979233. doi: 10.1177/2054358120979233.

87. Torres-Jimenez AR, Ramirez-Nova V, Cespedes-Cruz AI, Sanchez-Jara B, Velazquez-Cruz A, Bekker-Méndez VC, et al. Primary antiphospholipid syndrome in pediatrics: beyond thrombosis. Report of 32 cases and review of the evidence. *Pediatr Rheumatol Online J.* (2022) 20: 13. doi: 10.1186/s12969-022-00673-y.

88. Gonzalez-Nieto JA, Martin-Suarez I, Quattrino S, Ortiz-Lopez E, Muñoz-Beamud FR, Colchero-Fernández J, et al. The efficacy of romiplostim in the treatment of severe thrombocytopenia associated to Evans syndrome refractory to rituximab. *Lupus.* (2011) 20: 1321-1323. doi: 10.1177/0961203311404913.

89. Hadler BCM, and Borges H. Retinopatia vaso-oclusiva por lúpus eritematoso sistêmico associada à síndrome do anticorpo antifosfolipídeo. *Revista Brasileira de Oftalmologia.* (2018) 77: 50-53. doi: 10.5935/0034-7280.20180011.

90. Horikoshi M, Inokuma S, Matsubara E, Honda Y, Okada R, Kobuna M, et al. Atypical Subacute Recurrence of Catastrophic Antiphospholipid Syndrome in a Japanese Female Patient. *Intern Med.* (2015) 54: 2923-2927. doi: 10.2169/internalmedicine.54.5150.

91. Horita S, Zoshima T, Hara S, Koichi M, Hirayama S, Suzuki K, et al. Antiphospholipid antibody syndrome-associated renal thrombotic microangiopathy improved not with rivaroxaban but with warfarin in a systemic lupus erythematosus patient without lupus nephritis. *CEN Case Rep.* (2021) 10: 409-413. doi: 10.1007/s13730-021-00581-2.

92. Haselboeck J, Ringl H, Mueller C, Pabinger I, and Winkler S. Post-surgical hemorrhagic infarction of the adrenal gland as the first clinical manifestation of antiphospholipid syndrome after 43 years of antibody-positivity. *Modern Rheumatology.* (2013) 23: 1237-1241. doi: 10.1007/s10165-012-0741-7.

93. Lopetegui-Lia N, Asad SD, Jafri SI, and Harrison JS. Autoimmune Diseases and Rosai-Dorfman Disease Coexist More Commonly than Expected: Two Case Reports. *Am J Case Rep.* (2019) 20: 770-772. doi: 10.12659/ajcr.915627.

94. Mekala S, Kumar CGD, and Gulati R. Antiphospholipid Syndrome Complicating Pneumococcal Meningitis. *Indian Pediatrics.* (2018) 55: 429-431. doi: 10.1007/s13312-018-1289-1.

95. Kniaz D, Eisenberg GM, Elrad H, Johnson CA, Valaitis J, and Bregman H. Postpartum hemolytic uremic syndrome associated with antiphospholipid antibodies. A case report and review of the literature. *Am J Nephrol.* (1992) 12: 126-133. doi: 10.1159/000168432.

96. Barbour TD, Crosthwaite A, Chow K, Finlay MJ, Better N, Hughes PD, et al. Antiphospholipid syndrome in renal transplantation. *Nephrology.* (2014) 19: 177-185. doi: 10.1111/nep.12217.

97. Diószegi Á, Tarr T, Nagy-Vincze M, Nánásy-Vass M, Veisz R, Bidiga L, et al. Microthrombotic renal involvement in an SLE patient with concomitant catastrophic antiphospholipid syndrome: the beneficial effect of rituximab treatment. *Lupus.* (2018) 27: 1552-1558. doi: 10.1177/0961203318768890.

98. Scaramucci L, Giovannini M, Niscola P, Palombi M, Cupelli L, Tendas A, et al. IgA-induced autoimmune hemolytic anemia in a patient with antiphospholipid syndrome. *Asian J Transfus Sci.* (2012) 6: 188. doi: 10.4103/0973-6247.98950.

99. Núñez-Álvarez CA, Hernández-Molina G, Bermúdez-Bermejo P, Zamora-Legoff V, Hernández-Ramírez DF, Olivares-Martínez E, et al. Prevalence and associations of anti-phosphatidylserine/prothrombin antibodies with clinical phenotypes in patients with primary antiphospholipid syndrome: aPS/PT antibodies in primary antiphospholipid syndrome. *Thromb Res.* (2019) 174: 141-147. doi: 10.1016/j.thromres.2018.12.023.

100. Nagayama K, Izumi N, Miyasaka Y, Saito K, Ono K, Noguchi O, et al. Hemolysis, elevated liver enzymes, and low platelets syndrome associated with primary anti-phospholipid antibody syndrome. *Intern Med.* (1997) 36: 661-666. doi: 10.2169/internalmedicine.36.661.

101. Yousuf S, Junaid N, Adeboye A, Nazir H, Scatliffe K, Patel P, et al. A unique cause of pulmonary hemorrhage: A case of atypical hemolytic uremic syndrome in a patient with antiphospholipid antibody syndrome. *Chest.* (2017) 152: A889. doi: 10.1016/j.chest.2017.08.924.

102. Dhanawat A, Gupta P, Padhan P, and Mohanty L. Leg ulcers secondary to antiphospholipid syndrome in a young female. *Journal of Clinical and Diagnostic Research.* (2019) 13: OD06-OD07. doi: 10.7860/JCDR/2019/42988.13350.

103. Del Proposto G, Antonelli M, Cerrone P, Bruno A, Costantino L, Ricciardi E, et al. Cancer of the sigmoid colon and antibodies. A puzzle. *Transfus Apher Sci.* (2015) 52: 220-221. doi: 10.1016/j.transci.2014.12.025.

104. Abdelhalim O, Serna C, Guerrero N, Onasis I, and Abosheaishaa H. Secondary Evans Syndrome Presenting With Lupus Anticoagulant. *Cureus.* (2024) 16: e63992. doi: 10.7759/cureus.63992.

105. Asherson RA, Espinosa G, Menahem S, Yinh J, Bucciarelli S, Bosch X, et al. Relapsing catastrophic antiphospholipid syndrome: report of three cases. *Semin Arthritis Rheum.* (2008) 37: 366-372. doi: 10.1016/j.semarthrit.2007.08.001.

106. Espino AR, Ang MC, and Fortinez JT. Hughes syndrome preceding systemic lupus erythematosus for 8 years in a 30-year-old Filipino female patient: a case report. *Chest.* (2019) 155: 293A. doi: 10.1016/j.chest.2019.02.282.

107. Siu C, Delatorre M, Guthier D, and Hossain A. Secondary Evans syndrome with elevated antiphospholipid antibody titers. *Chest.* (2021) 160: A734. doi: 10.1016/j.chest.2021.07.696.

108. Gong YL, and Li YF. Anticardiolipin antibodies in concurrent poststreptococcal glomerulonephritis and autoimmune hemolytic anemia: A case report. *Arch Argent Pediatr.* (2018) 116: e288-e291. doi: 10.5546/aap.2018.eng.e288.

109. Lu Y, and Huang XM. Autoimmune hemolytic anemia as an initial presentation in children with systemic lupus erythematosus: two case reports. *J Int Med Res.* (2022) 50: 3000605221115390. doi: 10.1177/03000605221115390.

110. Sakaguchi S, Kitazawa K, Watanabe M, Mukai K, Totsuka D, Shibata T, et al. A case of primary antiphospholipid antibody syndrome with acute renal failure showing thrombotic microangiopathy. *Am J Nephrol.* (1999) 19: 594-598. doi: 10.1159/000013526.

111. Sivasailam B, and Feldman DM. Hepatic Infarction Associated Antiphospholipid Syndrome and HELLP in Pregnancy. *American Journal of Gastroenterology.* (2022) 117: S1953. doi: 10.14309/01.ajg.0000868716.82071.ba.

112. Arvieux J, Schweizer B, Roussel B, and Colomb MG. Autoimmune haemolytic anaemia due to anti-phospholipid antibodies. *Vox Sang.* (1991) 61: 190-195. doi: 10.1111/j.1423-0410.1991.tb00945.x.

113. Tkachenko O, Lapin S, Maslyansky A, Myachikova V, Mikhailova L, and Gilburd B. Relapsing Evans syndrome and systemic lupus erythematosus with antiphospholipid syndrome treated with Bortezomib in combination with plasma exchange. *Clin Immunol.* (2019) 199: 44-46. doi: 10.1016/j.clim.2018.12.010.

114. Papadopoulos KI, Jönsson A, Berntorp E, Törnquist C, and Hulthén UL. Primary antiphospholipid syndrome associated with postoperative primary adrenal failure. *J Intern Med.* (1995) 238: 175-178. doi: 10.1111/j.1365-2796.1995.tb00916.x.

115. Tanariyakul M, Nebrajas K, Saowapa S, and Polpichai N. Extensive Thrombosis in Catastrophic Antiphospholipid Syndrome in a Newly Diagnosed Systemic Lupus Erythematosus: A Case Report. *Cureus.* (2024) 16: e59542. doi: 10.7759/cureus.59542.

116. Suenaga A, Sawa N, Miki K, Yokoyama T, Ishii Y, Mizuno H, et al. Antiphospholipid Syndrome Nephropathy with Acute Thrombotic Microangiopathy after Renal Transplantation. *Internal Medicine.* (2023) 62: 2707-2713. doi: 10.2169/internalmedicine.0813-22.

117. Font J, López-Soto A, Cervera R, Balasch J, Pallarés L, Navarro M, et al. The 'primary' antiphospholipid syndrome: antiphospholipid antibody pattern and clinical features of a series of 23 patients. *Autoimmunity.* (1991) 9: 69-75. doi: 10.3109/08916939108997126.

118. Patel BP, and Jakob J. A Rare Case of Simultaneous Evans Syndrome and Primary Antiphospholipid Syndrome. *Cureus.* (2020) 12: e6845. doi: 10.7759/cureus.6845.

119. Vandenberghe P, Zachee P, Verstraete S, Demuynck H, Boogaerts MA, and Verhoef GE. Successful control of refractory and life-threatening autoimmune hemolytic anemia with intravenous immunoglobulins in a man with the primary antiphospholipid syndrome. *Ann Hematol.* (1996) 73: 253-256. doi: 10.1007/s002770050237.

120. Díaz-Cremades J, Fernández-Fuertes F, Ruano JA, Tapia M, Soler S, Bosch JM, et al. Concurrent thrombotic thrombocytopenic purpura and antiphospholipid syndrome: a rare and severe clinical combination. *British Journal of Haematology.* (2009) 147: 584-585. doi: 10.1111/j.1365-2141.2009.07856.x.

121. Koenig M, Roy M, Baccot S, Cuilleron M, de Filippis JP, and Cathébrasl P. Thrombotic microangiopathy with liver, gut, and bone infarction (catastrophic antiphospholipid syndrome) associated with HELLP syndrome. *Clinical Rheumatology.* (2005) 24: 166-168. doi: 10.1007/s10067-004-1017-1.

122. Fonseca E, Alvarez R, González MR, and Pascual D. Prevalence of anticardiolipin antibodies in subacute cutaneous lupus erythematosus. *Lupus.* (1992) 1: 265-268. doi: 10.1177/096120339200100411.

123. Gelman R, Kharouf F, Ishay Y, and Gural A. Cold Agglutinin-Mediated Autoimmune Hemolytic Anemia in Association with Antiphospholipid Syndrome. *Acta Haematol.* (2021) 144: 693-697. doi: 10.1159/000516295.

124. Raupov RK, Suspitsin EN, Imelbaev AI, and Kostik MM. Simultaneous Onset of Pediatric Systemic Lupus Erythematosus in Twin Brothers: Case Report. *Front Pediatr.* (2022) 10: 929358. doi: 10.3389/fped.2022.929358.

125. Mutter WP, Stillman IE, and Dahl NK. Thrombotic Microangiopathy and Renal Failure Exacerbated by ε-Aminocaproic Acid. *American Journal of Kidney Diseases.* (2009) 53: 346-350. doi: 10.1053/j.ajkd.2008.07.023.

126. Suzumori N, Obayashi S, Kumagai K, Goto S, Yoshida A, and Sugiura-Ogasawara M. A case of microangiopathic antiphospholipid-associated syndromes during pregnancy: Review of the literature. *Case Reports in Medicine.* (2012) 2012. doi: 10.1155/2012/827543.

127. Ruffatti A, De Silvestro G, Ghirardello A, Calligaro A, Del Ross T, Thiene G, et al. A catastrophic antiphospholipid syndrome: the importance of high levels of warfarin anticoagulation. *J Intern Med.* (1994) 235: 81-83. doi: 10.1111/j.1365-2796.1994.tb01036.x.

128. Nauseef JT, Lim HI, and DeSancho MT. Successful outcome with eculizumab treatment in a patient with antiphospholipid syndrome presenting with an unusual thrombotic storm. *J Thromb Thrombolysis.* (2021) 52: 597-600. doi: 10.1007/s11239-020-02343-w.

129. Paydas S, Koçak R, Zorludemir S, and Baslamisli F. Bone marrow necrosis in antiphospholipid syndrome. *J Clin Pathol.* (1997) 50: 261-262. doi: 10.1136/jcp.50.3.261.

130. Kolasinski SL. Prevalence of secondary hematologic disorders in the antiphospholipid syndrome: impact on coagulation risk. *Curr Rheumatol Rep.* (2006) 8: 95-99. doi: 10.1007/s11926-006-0048-9.

131. Chang PC, Chen WS, Lin HY, Lee HM, and Chen SJ. Combined central retinal artery and vein occlusion in a patient with systemic lupus erythematosus and anti-phospholipid syndrome. *Lupus.* (2010) 19: 206-209. doi: 10.1177/0961203309345751.

132. Sopeña B, Pérez-Rodríguez MT, Rivera A, Ortiz-Rey JA, Lamas J, and Freire-Dapena MC. Livedoid vasculopathy and recurrent thrombosis in a patient with lupus: seronegative antiphospholipid syndrome? *Lupus.* (2010) 19: 1340-1343. doi: 10.1177/0961203310373783.

133. Veskitkul J, Ruangchira-urai R, Charuvanij S, Pongtanakul B, Udomittipong K, and Vichyanond P. Asthma-like symptoms as a presentation of antiphospholipid syndrome. *Pediatric Pulmonology.* (2015) 50: E1-E4. doi: 10.1002/ppul.23079.

134. Baya W, Fredj FB, Hassine IB, Anoun J, Mzabi A, Karmani M, et al. Systemic lupus erythematosus, antiphospholipid syndrome and Hashimoto thyroiditis occurring in a patient with Niemann-Pick disease: a second case. *Pan Afr Med J.* (2020) 36: 367. doi: 10.11604/pamj.2020.36.367.25116.

135. de Holanda MI, Pôrto LC, Wagner T, Christiani LF, and Palma LMP. Use of eculizumab in a systemic lupus erythemathosus patient presenting thrombotic microangiopathy and heterozygous deletion in CFHR1-CFHR3. A case report and systematic review. *Clin Rheumatol.* (2017) 36: 2859-2867. doi: 10.1007/s10067-017-3823-2.

136. Vassia V, Croce A, Ravanini P, Leutner M, Saglietti C, Fangazio S, et al. Unusual presentation of fatal disseminated varicella zoster virus infection in a patient with lupus nephritis: A case report. *BMC Infectious Diseases.* (2020) 20. doi: 10.1186/s12879-020-05254-6.

137. Breccia M, D'Elia GM, Girelli G, Vaglio S, Gentilini F, Chiara S, et al. Paroxysmal cold haemoglobinuria as a tardive complication of idiopathic myelofibrosis. *Eur J Haematol.* (2004) 73: 304-306. doi: 10.1111/j.1600-0609.2004.00301.x.

138. Nauseef J, Lim H, Saab J, Magro C, and DeSancho M. Successful treatment with eculizumab in a patient with antiphospholipid syndrome presenting with a thrombotic storm. *Research and Practice in Thrombosis and Haemostasis.* (2019) 3: 682. doi: 10.1002/rth2.12229.

139. Demir S, Keskin A, Sağ E, Kaya Akca Ü, Atalay E, Cüceoğlu MK, et al. The challenges in diagnosing pediatric primary antiphospholipid syndrome. *Lupus.* (2022) 31: 1269-1275. doi: 10.1177/09612033221108853.

140. Rovere-Querini P, Canti V, Erra R, Bianchi E, Slaviero G, D'Angelo A, et al. Eculizumab in a pregnant patient with laboratory onset of catastrophic antiphospholipid syndrome: A case report. *Medicine (Baltimore).* (2018) 97: e12584. doi: 10.1097/md.0000000000012584.

141. Stanescu C, Andronesi AG, Jurcut C, Gherghiceanu M, Vornicu A, Burcea FA, et al. Successful Treatment of Catastrophic Antiphospholipid Syndrome Using Rituximab: Case Report and Review of the Literature. *Medicina (Kaunas).* (2021) 57. doi: 10.3390/medicina57090912.

142. Gallant TL, Zheng E, Hobbs AM, Becka AJ, and Bertsch RA. Complement-Mediated Thrombotic Microangiopathy in a Patient With Antiphospholipid Syndrome and Anti-glomerular Basement Membrane Antibodies. *Cureus.* (2023) 15: e42410. doi: 10.7759/cureus.42410.

143. Oulego-Erroz I, de Jubera JMS, Ocaña-Alcober C, Regueras-Santos L, Ferrero-De la Mano L, and Martínez-Badás JP. Pediatric Catastrophic Antiphospholipid Syndrome Successfully Treated with Eculizumab. *American Journal of Respiratory and Critical Care Medicine.* (2021) 203: 640-642. doi: 10.1164/rccm.202009-3489LE.

144. Hanai S, Kobayashi Y, Ito R, Harama K, and Nakagomi D. Thrombotic microangiopathy with refractory lupus nephritis successfully treated by combining rituximab with belimumab. *Scandinavian Journal of Rheumatology.* (2023) 52: 227-229. doi: 10.1080/03009742.2022.2140483.

145. Lazurova I, Macejova Z, Tomkova Z, Remenar F, Boor A, Lazur J, et al. Severe limb necrosis: primary thrombotic microangiopathy or "seronegative" catastrophic antiphospholipid syndrome? A diagnostic dilemma. *Clin Rheumatol.* (2007) 26: 1737-1740. doi: 10.1007/s10067-006-0487-8.

146. Nawata A, Shirayama R, Oshida K, Sato T, Ito T, Shiba E, et al. Catastrophic antiphospholipid syndrome with Epstein–Barr virus-associated hemophagocytosis: A clinicopathological conference. *Lupus.* (2022) 31: 1385-1393. doi: 10.1177/09612033221118819.
